# *Hypocrea jecorina *CEL6A protein engineering

**DOI:** 10.1186/1754-6834-3-20

**Published:** 2010-09-08

**Authors:** Suzanne E Lantz, Frits Goedegebuur, Ronald Hommes, Thijs Kaper, Bradley R Kelemen, Colin Mitchinson, Louise Wallace, Jerry Ståhlberg, Edmundo A Larenas

**Affiliations:** 1 Genencor Division, Danisco USA Inc., 925 Page Mill Rd. Palo Alto, CA 94304, USA; 2 Genencor, a Danisco Division, Archimedesweg 30, 2333CN, Leiden, The Netherlands; 3 Department of Molecular Biology, Swedish University of Agricultural Sciences, POB 590, SE-751 24 Uppsala, Sweden

## Abstract

The complex technology of converting lignocellulose to fuels such as ethanol has advanced rapidly over the past few years, and enzymes are a critical component of this technology. The production of effective enzyme systems at cost structures that facilitate commercial processes has been the focus of research for many years. Towards this end, the *H. jecorina *cellobiohydrolases, CEL7A and CEL6A, have been the subject of protein engineering at Genencor. Our first rounds of cellobiohydrolase engineering were directed towards improving the thermostability of both of these enzymes and produced variants of CEL7A and CEL6A with apparent melting temperatures above 70°C, placing their stability on par with that of *H. jecorina *CEL5A (EG2) and CEL3A (BGL1). We have now moved towards improving CEL6A- and CEL7A-specific performance in the context of a complete enzyme system under industrially relevant conditions. Achievement of these goals required development of new screening strategies and tools. We discuss these advances along with some results, focusing mainly on engineering of CEL6A.

## Background

*Hypocrea jecorina *(anamorph *Trichoderma reesei*) is an industrially important producer of cellulases for an applications portfolio that includes pulp and paper processing, food and feed processing, textile manufacturing and modification, and detergents/cleaners formulation. Cellulases were employed in early 'waste to transportation fuel' research in, for example, the U.S. Army Natick program in the 1970s; however, it was not until 2007 that a cellulase formulation for biomass saccharification was developed and marketed to the nascent biomass-to-ethanol industry (Accellerase^®^1000; Genencor Division, Danisco USA Inc.).

### Mechanism

To understand the challenges of protein engineering biomass enzymes, it is important to understand current concepts of their structure-function relationship. Early concepts of cellulase mode of action were published by Reese *et al. *in 1950 [1], followed by a review article in 1964 [2]. Since then, a great deal of work has been devoted to elucidating the mechanism of enzymatic cellulose degradation. These studies have covered a wide range of substrates from pure cellulose to pretreated lignocellulose from various sources [3-8]. Yet, there is still much to learn regarding the fundamental mechanism of cellulases.

Two of the most important cellulase glycosyl hydrolase families are GH6 and GH7. The first structure of a cellobiohydrolase to be solved was the catalytic module of *H. jecorina *CEL6A (previously known as CBH 2), which was published by Rouvinen *et al. *in 1990 [9]. The first structure of a fungal cellulose binding module, the CBM1 of *H. jecorina *CEL7A (previously known as CBH 1), was published by Kraulis *et al. *in 1989 [10], and a few years later the structure of the *H. jecorina *CEL7A catalytic module was published by Divne *et al. *in 1994 [11].

The catalytic mechanism of glycosyl hydrolase family 6 enzymes, including both exo- and endoglucanases is net inversion (versus retention in family 7) of the anomeric configuration(Carbohydrate Active Enzymes database http://www.cazy.org/; Cantarel [12]). Although there is no overall sequence homology between the catalytic domains of the *H. jecorina *cellobiohydrolases CEL6A and CEL7A, there is homology between CEL6A and GH6 endoglucanases and between CEL7A and GH7 endoglucanases. It is the differences, particularly in the loop regions that form the active site tunnel in CEL6A and CEL7A, that affect the type of enzyme activity. These loops are missing in the endoglucanases, leaving a more open active site cleft [9,11]. Indeed, deletion of the C-proximal loop that covers the active site of *Cellulomonas fimi *cellobiohydrolase A increased its endoglucanase activity [13]. Furthermore, Divne *et al. *[11] concluded that dissimilarities in the CEL6A and CEL7A tunnels (length, number of tryptophans, and asymmetric distribution of glucose binding subsites) could explain observations of distribution differences on cellulose fibrils and synergy between CEL7A and CEL6A.

Advanced modeling [14,15] and microscopy techniques [16,17] are being used to study cellobiohydrolase-substrate interactions. These techniques hold promise for demonstrating how these enzymes bind substrate and move and how these events are related to hydrolysis. Studies of the *H. insolens *CEL6A [18] provided a detailed description of solvent-mediated carbohydrate-protein interactions involved in ligand movement through the active site tunnel during hydrolysis using crystal structures to support the authors' model. Electron microscopy studies with enzymes from *Humicola insolens *confirmed that the GH6 and GH7 cellobiohydrolases act processively from opposite ends of the cellulose chain, GH6 from the nonreducing end and GH7 from the reducing end [19]. These studies also revealed that CEL6A had substantial endoglucanase activity and lower processivity than CEL7A, at least on the Valonia cellulose crystals studied. However, on a complex biomass substrate, in a natural mix of cellulases, it is not known to what extent CEL6A relies on its own endoglucanase activity and to what extent it starts from chain ends created by other endoglucanase enzymes. The Walker lab at Cornell University uses single-molecule detection methods, such as quantitative fluorescence microscopy, to study cellulase adsorption and hydrolysis [20]. Recently, researchers have observed cellulases sliding along a cellulose fibril in real time and measured the velocity of sliding [21]. While the basic chemical mechanisms of glycosyl hydrolases are well understood and their combined roles in the hydrolysis of pure crystalline cellulose are reasonably well elucidated, hydrolysis of lignocellulosic substrates is more complicated and is the subject of continuing research. Much of the applied research is now directed towards discovering the minimum number of enzyme components that provide optimal performance.

### Optimal cellulase mixture for biomass hydrolysis

To date, optimal mixtures have generally been developed by purely empirical approaches [22,23] and may vary relative to the substrate being used. The CEL7A and CEL6A cellobiohydrolases are the two most abundant enzymes in *H. jecorina *[23], indicating their key role in the cellulase enzyme system. The importance of CEL7A and CEL6A was demonstrated in 1980 by Reese and Mandels [24] when they showed that cellobiohydrolase activity was limiting cellulose hydrolysis. They also demonstrated that the cellobiohydrolase component was less stable under standard process conditions (pH 4.8, 50°C, 24 hr) than most of the other enzymes in the native *H. jecorina *cellulase milieu. That the cellobiohydrolases are indeed key players was further supported by individual deletion of the four major cellulase genes *cbh1, cbh2, egl1 *and *egl2 *(coding for CEL7A, CEL6A, CEL7B and CEL5A) in the industrial *H. jecorina *strain VTT-D-79125, where the mutants without CEL7A or CEL6A showed a 70% and 33% activity loss, respectively, on filter paper relative to the parent strain [25]. This study, and that of Reese and Mandels [24], was conducted with pure cellulose substrates (filter paper and Avicel, respectively). Rosgaard *et al. *[23], using two different hot water-pretreated barley samples, demonstrated optimal performance when the CEL6A:CEL7A ratio was 2:1, again showing that cellobiohydrolase activity exceeding that of native *H. jecorina *preparations was beneficial.

The issues of inhibition (substrate and product) and substrate accessibility in these complex mixtures have been recognized for some time [26-29]. It was also accepted early on that efficient cellulose-digesting enzymes would need to be produced with acceptable economics. Much effort has been made to improve the productivity of strains descending from the original *T. reesei *QM6a [30]. Programs such as the NTG mutagenesis and selection conducted at Rutgers University [31] and Lehigh University [32] were quite successful and resulted in strains that are still in industrial use, such as RutC30 and RL-P37, and their descendants. Strain improvement has continued even with the relatively high productivity of these industrial strains. To date, enzyme cost reduction has been mainly accomplished by strain improvement rather than by protein engineering.

### Protein engineering

The generally low turnover rates of cellulases, for example, one to four per second for Cel7A [33], present a challenge in applications where cheap sugars are the desired product. Faster rates may be accomplished by raising the reaction temperature and/or by increasing the specific activity of the enzyme. CEL6A has been the target of several protein engineering efforts; some were described in Schülein's 2000 review [34] of cellulase engineering, where he acknowledged that most of the work was devoted to understanding the catalytic mechanism. At VTT, Koivula *et al. *[35] identified the CEL6A catalytic domain surface residue W272 as essential for degradation of crystalline cellulose, but not soluble or amorphous cellulose. Wohlfahrt *et al. *[36] identified carboxyl-carboxylate pair mutations (to the corresponding amide-carboxylate pair) to be useful in stabilizing *H. jecorina *CEL6A, particularly with respect to pH. More site-directed mutations pointed to a role for Y169 in kinetics and binding [37]. The Y169F mutation resulted in little change in the crystal structure relative to wild-type Cel6A, but the association constants for cellotriose and cellotetraose increased fourfold while the activity decreased to about one fourth its original level. The authors speculate that the Y169 residue imposes a distortion of glucose to a more reactive conformation in the active site tunnel. Mutations of two carboxylic acid residues, D175 and D221, at the catalytic centre of CEL6A, supported the proposed role of D221 as responsible for protonation of the glycosidic oxygen, while molecular dynamics simulations indicated that D175 indirectly fulfills the role of a catalytic base [38]. However, in most of the structures of GH6 enzymes, D221 (or the equivalent residue) makes a hydrogen bond to and shares a proton with D175; therefore, D221 cannot protonate the glycosidic oxygen. Formation of a catalytically competent configuration seems to be associated with closing of a flexible loop in association with the substrate, which isolates two water molecules at the catalytic centre [39]. D175 is in contact with and may accept a proton from one of the water molecules, which may in turn accept a proton from the second water that is in position for nucleophilic attack on the anomeric carbon of the sugar. But the closing of the loop also restricts the passage between successive glucose binding subsites within the active site, suggesting that processive action along a cellulose chain requires a cyclic closing-opening sequence for each hydrolytic event, in conformity with molecular dynamics [38]. Flexibility in this loop may thus be an important factor for the specific activity of the enzyme.

Wilson and colleagues [40-42] have worked extensively with *Thermobifida fusca *CEL6B exocellulase and have characterized the catalytic mechanism using enzyme variants. With CEL6B variants expressed in *S. lividans *or *E. coli*, they demonstrated that lower activity with insoluble substrates was linked to reduced processivity and that adding disulfide bonds across the loops forming the active site tunnel reduced ligand binding, processivity and activity. In addition, they identified noncatalytic CEL6B mutations in which single and double mutants (G234 S, G284P) demonstrated higher activity on swollen cellulose and filter paper, but these improved variants did not increase synergism with the *T. fusca *endoglucanase Cel5A [40]. Mutations near the substrate binding site were found to reduce cellobiose inhibition, but in most cases (except G234S) the mutations also resulted in reduced thermal stability. The effect of cysteine residue mutations on expression and thermostability was related to the position of the residue(s) and whether it led to aberrant disulfide bond formation, improper folding, and sometimes proteolysis [41]. Wilson described these and other complexities of engineering cellulases for enhanced activity in a 2009 review article [42]. The review cautions that (1) there is a dearth of demonstrated cellulase activity improvement, (2) improving a single activity may be irrelevant if performance of the synergistic mixture is not improved, (3) protein engineering using only the catalytic module does not guarantee the same performance in the full length protein and (4) activity improvement demonstrated on one substrate does not guarantee the same results on a different substrate.

Recently, Heinzelman and colleagues [43] demonstrated that CEL6A chimeras, which included sequences from *H. jecorina *CEL6A, CEL6A from the thermophilic fungus *Humicola insolens*, and/or sequences from three other fungi, expressed by *S. cerevisiae*, resulted in molecules with greater thermostability than either parent. The chimeras demonstrated a broader pH range than *H. jecorina *CEL6A. Of the chimeras described, none exceeded CEL6A specific activity on phosphoric acid swollen cellulose (PASC). In a related study [44], they identified a single mutation that significantly enhanced *H. jecorina *CEL6A thermal stability with PASC hydrolysis activity similar to wild type.

Yet, after all these years, cellulosic ethanol is still expensive relative to starch ethanol. The saccharifying enzymes compose a significant cost component, so it is our objective to reduce the dose required to convert pretreated biomass to simple sugars for fermentation. One way to optimize performance is through protein engineering. Engineered cellulases have been used in textile applications, but few examples exist in the field of biomass saccharification. Unlike textile enzyme products which often are monocomponent, lignocellulosic biomass conversion requires a complex mixture of enzymes. This presents several challenges to a protein engineering approach to increasing performance. For example, it is not efficient to engineer all of the required enzymes. However, within the enzyme complex used for lignocellulosic conversion, some activity is always going to be either limiting or required in abundance. The preferred protein target(s) for improvement is an enzyme that is limiting and has the potential for making a large impact on the specific performance of the system. This improvement could be the result of one or a combination of improvements such as increased specific activity, reduced product inhibition, reduced nonproductive binding, or enhanced stability and longevity under process conditions. Studies using pure cellulose and traditional methods for detecting sugar release [45-49] have provided insight into enzyme mode of action, but pure cellulose does not necessarily predict enzyme performance on pretreated lignocellulose. Such improvements should be measured under process-relevant conditions, making development of the appropriate screening assay critical. Screens conducted in the complex biomass milieu will enable the detection of variants that are less susceptible to inhibition and inactivation from components found in the biomass of interest.

## Results and Discussion

### Mixture studies

**Figure 1:**
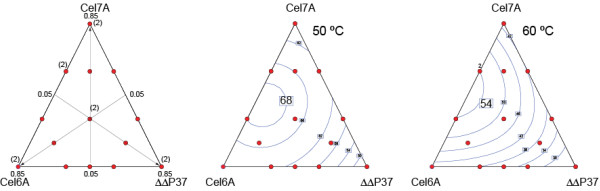
**Mixture experiment**. Three component design of experiment shows limitation and thermolability of cellobiohydrolase activity compared to the cellulase background of delta-CEL7A delta-CEL6A RL-P37. Shown are the experimental design points (left), contour plots for 50°C saccharification (middle) and 60°C saccharification (right). Red dots represent actual data points. Duplicate data points are indicated by the label (2). Contour labels indicate percent glucan conversion.

We demonstrated that the performance optima of a cellulase mixture also favoured more cellobiohydrolase activity using a process-relevant substrate, dilute acid-pretreated sugarcane bagasse, in a computer-designed mixture experiment (Design Expert Dx5). In this experiment, purified *H. jecorina *CEL7A and CEL6A were combined in various ratios with an RL-P37 background sample from which the *cel7A *and *cel6A *genes had been deleted (delta delta P37). Samples were dosed such that the final cellulose concentration of the mixture was 6% (wt/wt). In the three component mixtures, the amounts of CEL7A, CEL6A and delta delta P37 proteins ranged from 5 to 85 weight percent of the total mixture protein. Five percent (wt/wt) *H. jecorina *Cel3A (beta glucosidase 1) was included in all of the mixtures to mitigate the impact of cellobiose inhibition by converting most of the soluble cellooligosaccharides to glucose, which was analyzed by high-pressure liquid chromatography (HPLC). At both 50°C and 60°C, the performance of the mixture favoured more CEL7A and CEL6A activity and in approximately equal proportions, whereas in the native preparation CEL7A is three- to fourfold more abundant than CEL6A (Figure 1). The cellobiohydrolases were more limiting at 60°C than at 50°C, possibly due to their relatively lower thermostability. Enhanced activity and thermal stability of cellobiohydrolases are thus target properties to enhance for improvement of biomass conversion performance.

### Cellulase engineering for thermostability

From 2000-2004, Genencor worked with the National Renewable Energy Laboratory (NREL) under the auspices of the Department of Energy Office of the Biomass Program and improved the thermal stability of CEL6A and CEL7A through protein engineering.

Differential scanning calorimetry (DSC) was used to determine the thermal midpoint (*T*_m_) values of the thermal unfolding process for the most abundant *H. jecorina *cellulases and β-glucosidase (Table 1). Excessive heat capacity curves were measured using an ultrasensitive scanning high-throughput microcalorimeter, VP-Cap DSC (MicroCal, Inc., Northampton, MA). *H. jecorina *enzymes (500 μl of 0.5 mg/ml) were scanned over 30-90°C temperature range in 10 mM sodium acetate buffer, pH 5.0. A 200°C/hr scan rate was used. The *T*_m _of the DSC curves was used as an indicator of the thermal stability and calculated using the Origin Lab 7.0 software. Native CEL6A and CEL7A are more thermolabile than CEL5A and CEL3A, which may affect whole cellulase performance in extended saccharification reactions at >50°C. With the screening tools available in 2000, protein engineering was undertaken to improve the thermal stability of the two *H. jecorina *cellobiohydrolases.

**Table 1:** *H. jecorina *cellulases.

Enzyme	Class	Wt/wt%	*T*_m _(°C)
CEL7A (CBH I)	EXO	40-60	61.2
CEL6A (CBH II)	EXO	12-20	67.2
CEL7B (EGL I)	ENDO	5-10	67.6
CEL5A (EGL II)	ENDO	1-10	72.5
CEL12A (EGL III)	ENDO	< 1-5	63.0
CEL3A (BGL1)	β-glucosidase	1-2	77.0

Relative abundance [23] and *T*_m _values for the thermal unfolding process measured by differential scanning calorimetry in 10 mM acetate buffer, pH 5.0, of *H. jecorina *cellulases and β-glucosidase are shown.

Different mutagenesis and screening approaches were used for CEL7A and CEL6A *T*_m _improvement. The melting point of CEL7A was increased through a combination of random and site-directed mutagenesis and screening (US 2007/0173431). A limited number of sites with potential involvement in stability were selected on the basis of structure and a 42-member CEL7A sequence alignment of *Hypocrea *and *Trichoderma *family members. Site saturation mutagenesis was performed on these sites. CEL7A variants containing from 1 to 19 mutations were expressed in *A. niger *for screening, and approximately 100,000 clones were assayed for improved stability. Stability was determined by the difference in 4-methylumbelliferryl-lactoside (Sigma Chemicals, M2405) activity before and after a heat challenge. Select *A. niger*-expressed variants were purified by hydrophobic interaction chromatography, and thermal stability was determined by circular dichroism spectrophotometry (CD) [50-52].

Point mutations were then combined to obtain CEL7A variants with substantially higher thermal stability (Goedegebuur *et al.*, manuscript in preparation). Eighteen sites were combined to produce one CEL7A variant, which when expressed in *T. reesei *had a *T*_m _increase of 14.8°C (*T*_m _76.0°C) as determined by DSC. While screening was performed on *A. niger*-expressed proteins, lead molecules were then expressed in *T. reesei *for validation of performance, expression, and *T*_m _determination.

CEL6A variants were also expressed in *A. niger *for screening. A limited number of sites were selected for mutagenesis through a consensus approach (US 20060205042). A sequence alignment of *H. jecorina *CEL6A and eight GH6 family members was used to construct a consensus sequence. Single and multiple amino acid mutations were designed and made by site mutagenesis. Nonconserved positions were examined in the crystal structure, and mutations were selected that were different in CEL6A from the consensus and which fit the structure without disturbance. Conserved sites were not changed. More than 5000 clones were screened on PASC (prepared from Avicel PH101 [53]) for remaining activity after heat inactivation for 1 hr at 61°C or 65°C at pH 4.85. Combinations of mutations were made and ultimately resulted in an *H. jecorina*-expressed CEL6A variant with a *T*_m _increase of 6.9°C (as determined by DSC). This thermostable variant was shown to have similar activity to the CEL6A wild type in a reconstituted whole cellulase in dilute acid pretreated corn stover (PCS [54]) hydrolysis. PCS (7% wt/wt cellulose) specific performance was tested at 53°C for 20 hr by adding CEL6A variants to a CEL6A-deleted cellulose strain product (US 2006/0205042). These two protein engineering projects resulted in apparent melting temperatures above 70°C for both CEL7A and CEL6A, placing their stability on par with that of CEL5A and CEL3A.

### Cellulase engineering for performance

With this foundation of knowledge and experience, and with new tools in hand, we tackled the challenge of improving the specific activity of *H. jecorina *CEL6A. The products of this research will contribute to the cost reduction of enzymes in biomass-to-ethanol processes and one example for the application of the improved enzymes is the demonstration plant that the DuPont Danisco Cellulosic Ethanol, LLC (DDCE) began operating in early 2010.

Since our initial protein engineering work with NREL, we have developed *T. reesei *as a screening host for protein engineering and made other technical advancements on the basis of the lessons learned during those early years. Because *T. reesei *is a preferred host for enzyme production, we developed it as the host for screening to ensure that expression and performance of the selected variants would not be lost when expressed in the production host. The requirements for an effective high throughput screening strain include high frequency of transformation, reliable gene expression, reproducible growth in microtiter plate (MTP) format, sufficient and reproducible protein production, and low secretion of background proteins. The latter was especially important because the candidates for protein engineering were overexpressed using a cellulase induction system that had the potential to induce production of confounding background activities. The strain basis for screening was a fourfold deletion variant of *T. reesei *in which the genes for *cel7A, cel6A, cel7B*, and *cel5A *had been removed.

**Figure 2:**
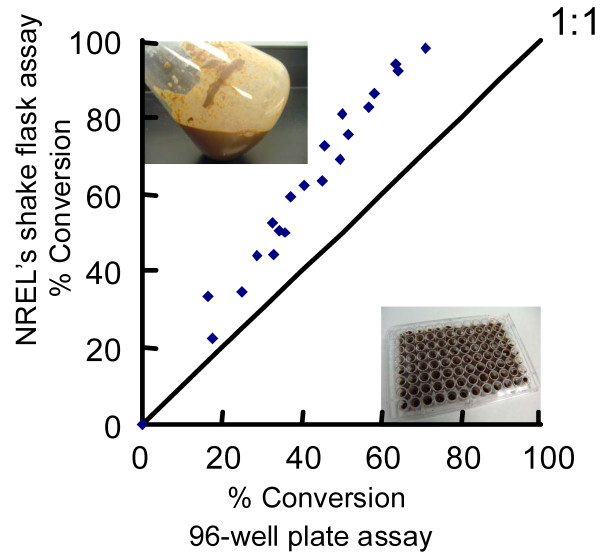
**Saccharification assays**. The miniaturized saccharification assay with dilute acid pretreated corn stover is predictive of shake flask scale performance. The shake flask assay was conducted by NREL according to their Laboratory Analytical Procedure (LAP) "Enzymatic Saccharification of Lignocellulosic Biomass" http://www.nrel.gov/biomass/pdfs/42629.pdf.

The biomass saccharification assay was miniaturized from shake flasks to 96-well MTP. Although miniaturized, the assays incorporated process relevant conditions. Either washed or unwashed pretreated biomass was used as the substrate. The substrate was delivered to the MTP as slurry in pH 5 sodium acetate buffer, with a consistency like cake batter. We demonstrated that the MTP scale assay was predictive of shake flask scale results (Figure 2). We also found that the MTP scale assay was predictive of larger-scale performance (not shown). MTP and shake flask saccharification assays were incubated for 3-5 days at 50°C, with shaking, using washed PCS at 13% wt/wt final solids (7% wt/wt cellulose). The correlation was shown by comparison of MTP scale results with shake flask scale results from NREL using the same materials and conditions http://www.nrel.gov/biomass/pdfs/42629.pdf. Each scale presented different challenges in delivery, mixing, and sampling. It is critical that the small-scale assays predict large-scale results. Screens performed with pure cellulosic substrates are often not predictive and do not allow for a mechanistic understanding of complex substrates. In fact, the results of screening with a particular complex substrate may not accurately predict performance on a different complex substrate. This is illustrated in the results of a performance comparison of 62 independent samples of *T. reesei *whole cellulase in saccharification assays with four different substrates: dilute acid pretreated sugarcane bagasse, PCS, Avicel, and PASC. The dilute acid PCS and bagasse were produced and provided by NREL [54]. The composition of lignocellulosic materials was determined using the assays detailed in the NREL protocols for Standard Biomass Analytical Procedures http://www1.eere.energy.gov/biomass/analytical_procedures.html.

**Figure 3:**
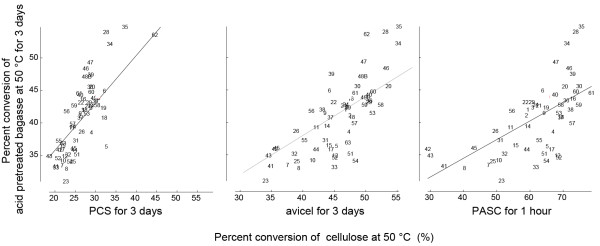
***H. jecorina *cellulase performance**. Comparison of saccharification performance (glucan conversion) on biomass and model cellulosic substrates.

The 62 cellulase samples represented material from various Genencor *T. reesei *strains, production lots, protein production conditions, and formulations, collected over several years. In the performance assays, the cellulases were dosed at 20 mg total protein per gram of cellulose. Total protein was determined by an automated Biuret method (Pointe Scientific T7528). The substrate loading of the PCS, bagasse, and Avicel was 7% (wt/wt) cellulose. Substrates were incubated with the cellulases for 3 days at 50°C, pH 5, and 200 rpm shaking. The cellulases were incubated with 1% (wt/wt) PASC at 50°C, pH 5, with shaking for 1 hr. Cellulose hydrolysis was measured either by a reducing sugar release assay (e.g., PAHBAH assay [49]), or by HPLC. Although clean cellulose such as Avicel and PASC generally correlated with lignocellulose conversion, there were exceptions (Figure 3). For example, sample 35 showed overall good performance on all four substrates; however, sample 57 performed well on PASC and Avicel and poorly on bagasse and PCS. Overall, enzyme performance was greater on bagasse than on PCS. There was little correlation between performance and enzyme production process or formulation or sample age. On the basis of previous experience, all samples were assumed to be stable during storage.

Because of these results, our screening assays for protein engineering were developed to bring them closer to actual use conditions. This required the development of biomass performance screens in which the activity of a specific enzyme or variant could be queried within a cellulase background. The challenge of screening for the target cellulase activity in a background of other cellulases is not trivial due to the synergistic nature of the enzymes. Another advance was using pretreated lignocellulosic substrate at high solids. Screening for CEL6A specific activity improvements required development of two high-throughput assays: one that showed dose dependence with respect to CEL6A concentration and one to accurately determine the concentration of expressed CEL6A. Variants were screened in a reconstituted cellulase background lacking cellobiohydrolase activity and including sufficient β-glucosidase activity to produce primarily glucose, which was measured by reducing sugar analysis using the PAHBAH method [49]. The substrate was washed PCS. Specific activity screening became possible with HPLC determination of protein concentrations in a 96-well MTP format. Although specific activity was the target property for improvement, stability using PASC was also measured.

Our protein engineering approach was to create Site Evaluation Libraries (SELs) that contained all 19 amino acid substitutions (including recreation of wild type). The libraries were generated in *E. coli*, variants were sequenced, and plasmid DNA was transformed into *T. reesei *for expression and screening. Each variant was screened for multiple properties to ensure that important properties, such as thermal stability and performance, were not lost.

Selection of the CEL6A sites for engineering was based on knowledge of the enzyme structure and guided by sequence alignments. More than 100 nonconserved CEL6A residues were selected for mutagenesis. They covered about 30% of the molecule concentrating on catalytic domain surface residues, but also including sites in the linker region and the carbohydrate binding module. Active site residues were not targeted in this study.

**Figure 4:**
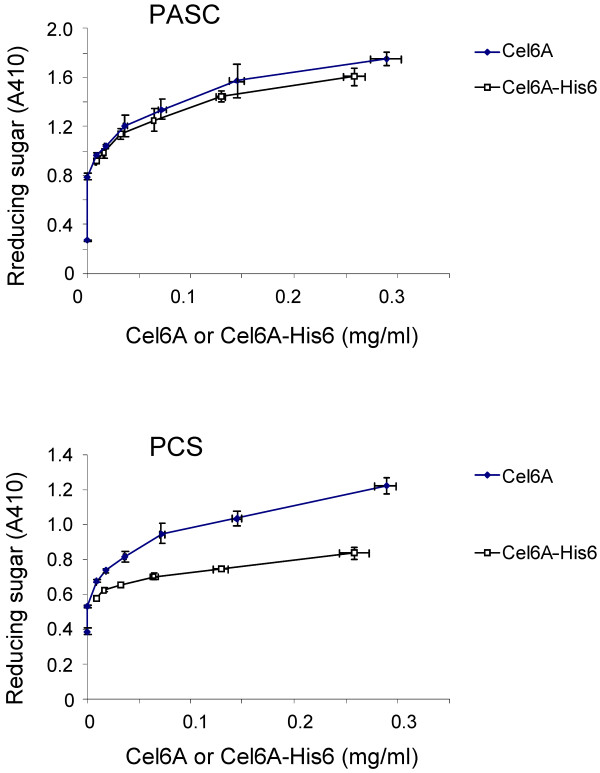
**Relative activity of CEL6A molecules**. Purified CEL6A and purified CEL6A-His6 were compared in two cellulose hydrolysis activity assays. CEL6A and CEL6A-His6 exhibited similar activity in PASC hydrolysis (top graph). PASC activity was determined by measurement of reducing sugars by PAHBAH following 1-hr incubation at 50°C in 1% (wt/wt) PASC. However, CEL6A-His6 activity was compromised, compared to native CEL6A, in hydrolysis of dilute acid pretreated corn stover (bottom graph), PCS miniaturized assay.

Although it was tempting to use a tagged molecule for ease of separation of the protein of interest from the cellulase background, we demonstrated that a C-terminal His tag on CEL6A caused reduced performance in biomass assays. In contrast, PASC assay performance of CEL6A was not affected by the tag, which emphasized again the need to use process-relevant conditions in screens (Figure 4). Instead, we developed proprietary high-throughput assays for HPLC determination of CEL6A and variant protein concentration to enable calculation of specific activities and dose-dependent biomass performance of CEL6A.

**Figure 5:**
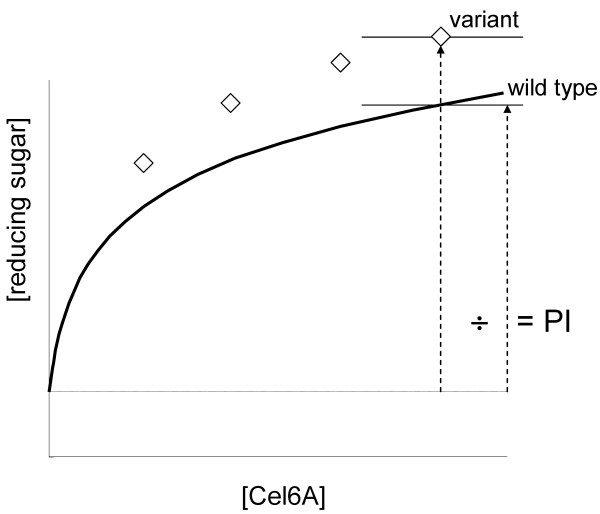
**Performance index**. The performance index is the ratio of performance of the variant to the wild-type protein. PI >1 is improved over wild-type performance.

**Figure 6:**
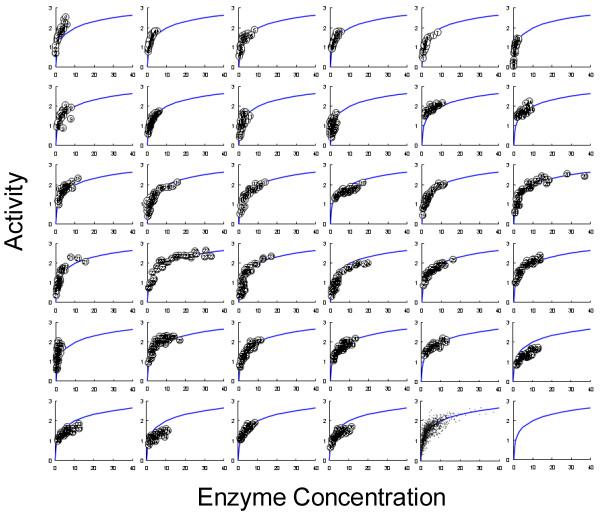
**PASC assay controls**. The sugar detected from the addition of the wild-type control CEL6A on a plate-by-plate basis plotted with a global curve fit (shown in the bottom right graph) to all of the data (second from the right at the bottom) collected during screening.

MTP-scale saccharification assays using PCS and PASC were used to screen the CEL6A variants. Serial dilutions of the CEL6A variants were added to the PCS saccharification assay such that a dose-response curve could be generated. Cellulose hydrolysis was determined by measurement of the increase in reducing sugars (PAHBAH). A performance index (PI) was calculated for each variant. The performance index is the ratio of performance of the variant to the wild-type protein. Generally an improved variant would have a PI >1, as shown in Figure 5. Although saccharification data is not linear with respect to CEL6A concentration and requires a curve fit, improved variants can be detected with this assay and analysis. CEL6A controls were included in each MTP in two formats. Plate-to-plate reproducibility of wild-type CEL6A was compared for PASC performance and found to be acceptable for detecting winners. Each growth plate contained recreated wild types in the SELs as well as wild-type controls. In Figure 6, each graph shows the activity of the wild-types on each PASC assay plate plotted against the isotherm fit for the activity of the wild-types from all 34 plates (blue line). The same comparison was made for PCS and found to be acceptable. In addition to the PCS and PASC saccharification assays, CEL6A variants were also screened for ethanol stability and heat stability (PASC activity before and after heating at a challenge temperature).

**Figure 7:**
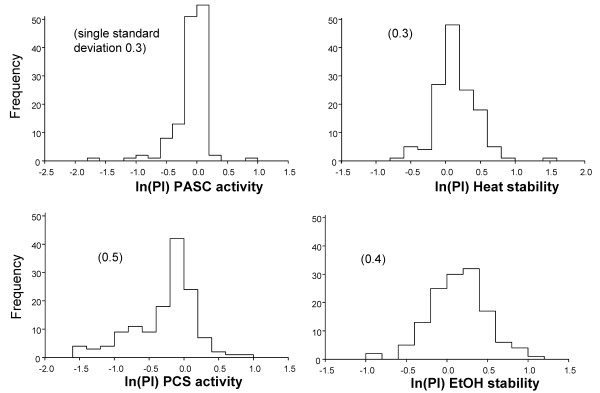
**Normal distribution of CEL6A controls**. Activity values of the library recreated wild-type CEL6A, not used for curve fitting, were transformed to determined PI values and further transformed with the natural log to obtain data that is normally distributed.

For each assay, we graphed the natural log of the PI for wild-type performance (those that were recreated within each site library). The transformed data are a Gaussian distribution (Figure 7) centered on zero for each property. These wild-type transformants were not used to calculate the PI. Two types of wild-type transformants were on each library plate: those that were used to calculate the PI (the controls) and the recreated wild types that were used to test the curve fit. The Gaussian distribution of the wild types was as expected.

**Figure 8:**
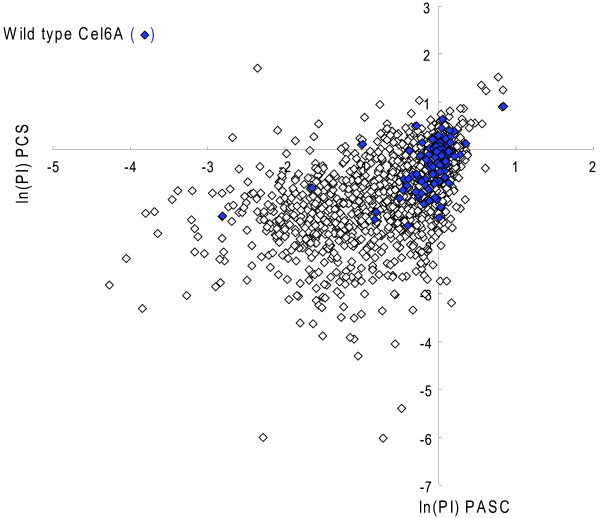
**Activity assay results**. All of the wild-type and CEL6A variant activity data from the PASC and PCS assays was plotted. A correlation is observed between the two activity assays. Improved variants in both assays are observed in the upper right quadrant. Wild type is shown in blue diamonds.

**Figure 9:**
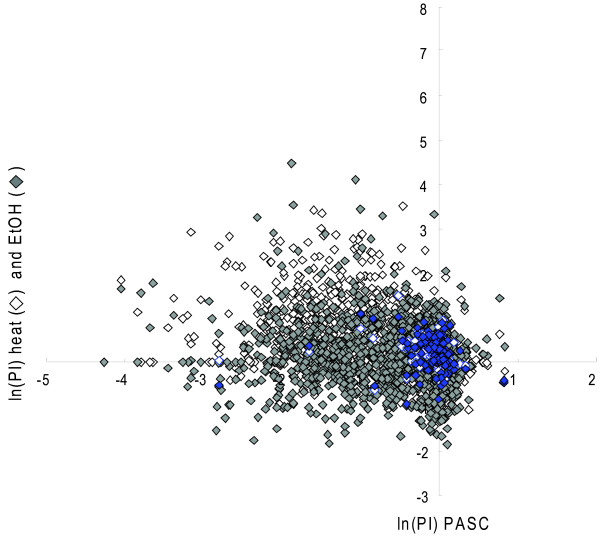
**Stability assay results**. All wild-type and CEL6A variant activity data from the PASC assay and both stability assays are plotted. Little correlation is observed between activity and stability. Improved variants in both properties are observed in the upper right quadrant. Wild type is shown in blue diamonds.

A correlation was observed between the two activity assays, PCS and PASC, from the graph of the natural log of the PI for all of the transformants (wild type and variants) (Figure 8). The variants in the upper right quadrant were improved approximately 2.5-fold over wild type. One false-positive wild type was observed in this quadrant. A correlation was also observed between the two stability assays: heat and ethanol (data not shown). There was little correlation between performance in the PASC activity assay and either stability assay (Figure 9).

The performance data can be sorted in a variety of ways, depending on the query objective. The most stringent case would be selection of mutations that resulted in improvements over wild type in all four assays (ethanol stability, heat stability, PASC activity and PCS activity). A less stringent selection would be for wild-type performance in some assays and improved performance in others. Several sites identified in previous CEL6A engineering efforts were included in the libraries and were identified again from the screen results.

## Conclusions

Biomass-to-ethanol plants are being constructed and operated today, but the foundation was laid by Mandels, Reese and others in the 1950s and 1960s. In fact, Reese credits Mandels with changing the focus of cellulase research at the U.S. Army Natick Laboratory from 'prevention of decomposition to promotion of decomposition' [55]. Reese and Mandels [24] demonstrated that cellobiohydrolase activity was limiting cellulose hydrolysis, but 30 years later, there are few reported successes in improving cellobiohydrolase-specific activity. There are many technical challenges to increasing cellulose-specific performance, including development of representative and predictive screens, expression of variants in an appropriate host, measurement of specific activity which includes high-throughput specific protein determination, and the ability to query cellulase activity in a background of confounding activities. *T. reesei *was demonstrated to be an effective high-throughput screening host for protein engineering. Specific activity screens were developed and shown to detect improvements in CEL6A activity in a *T. reesei *background with real biomass substrates at intermediate solids loadings. We improved the thermal stability of the two *H. jecorina *cellobiohydrolases to the same level as CEL5A and CEL3A. CEL6A variants were identified with higher activity than wild type and without loss of thermal stability. Since the methods used reflect process relevant conditions, they will help to quickly translate screening success to industrial success, without the potential pitfalls of changing substrate, solids loading, expression host, or protein background. While cost reductions will be achieved through process optimization, improved cellulases and cellulase preparations will be needed to further reduce the cost of delivering cheap sugars to the biofuels and biochemicals industries.

## Abbreviations

CBH: Cellobiohydrolase; DDCE: DuPont Danisco Cellulosic Ethanol, LLC; DSC: Differential Scanning Calorimetry; EG: Endoglucanase; MTP: Microtiter plate; NREL: National Renewable Energy Laboratory; PASC: Phosphoric acid swollen cellulose; PCS: Dilute acid pretreated corn stover; PI: Performance Index; SEL: Site Evaluation Library.

## Competing interests

The authors declare that they have no competing interests.

## Authors' contributions

SEL conducted cellulase performance assays and mixture experiments, FG developed assays and selected protein engineering sites, BRK developed assays and conducted mixture experiments, TK conducted His-tag comparison and selected engineering sites, CM selected engineering sites and provided technical leadership and coordination, RH coordinated and performed high throughput screening, LW performed DSC analysis and selected engineering sites, JS solved protein structures and selected engineering sites, EAL developed assays and conducted mixture experiments. All authors read and approved the final manuscript.
